# When oncogenes do not cause cancer

**DOI:** 10.7554/eLife.74912

**Published:** 2021-12-09

**Authors:** Jessica Shiu, Arthur D Lander

**Affiliations:** 1 Department of Developmental and Cell Biology, University of California, Irvine Irvine United States; 2 Department of Developmental and Cell Biology and the Center for Complex Biological Systems, University of California, Irvine Irvine United States

**Keywords:** melanoma, melanocytes, nevi, microRNA, mutation, Human

## Abstract

Environmental cues, not oncogene-induced senescence, may stop melanocytes with an activating mutation in the *BRAF* gene from turning into melanoma.

**Related research article** McNeal AS, Belote RL, Zeng H, Urquijo M, Barker K, Torres R, Curtin M, Shain AH, Andtbacka RH, Holmen S, Lum DH, McCalmont TH, VanBrocklin MW, Grossman D, Wei ML, Lang UE, Judson-Torres RL. 2021. BRAFV600E induces reversible mitotic arrest in human melanocytes via microRNA-mediated suppression of AURKB. *eLife* **10**:e70385. doi: 10.7554/eLife.70385

Cancers are a group of diseases in which an accumulation of mutations drives cells to multiply uncontrollably, and eventually spread to other parts of the body. However, a growing number of studies have shown that acquiring a single oncogenic (that is, cancer-driving) mutation does not always cause proliferation (Adashek et al., 2020). In some cases, cells remain unaffected or they may stop growing: some may even die (Martincorena et al., 2015; Damsky and Bosenberg, 2017; Brown et al., 2017; Schroeder et al., 2013).

For example, moles, or melanocytic nevi, emerge when melanin-producing cells called melanocytes start to proliferate. This is usually caused by an activating mutation in the *BRAF* gene – which is also one of the most frequent mutations found in a type of skin cancer known as melanoma (Damsky and Bosenberg, 2017). But although moles are extremely common, they nearly always stop growing on their own, and only rarely develop into melanoma.

Scientists have long believed that a process called oncogene-induced senescence explains why cells with oncogenic mutations stop growing (Serrano et al., 1997). The process is thought to be cell-intrinsic – a stress response triggered by abnormal signaling in the affected cell that permanently shuts down proliferation. Introducing mutated *BRAF* into skin melanocytes grown in the laboratory indeed causes the cells to stop dividing and turn on markers of senescence, but recent studies have challenged the view that this happens in moles. For example, not all nevus melanocytes display senescence markers, and some can resume growing after long periods of arrested division (Cotter et al., 2007; King et al., 2009; Ruiz-Vega et al., 2020).

Now, in eLife, Robert Judson-Torres and colleagues at the University of Utah, the University of California, San Francisco and the San Francisco Veterans Affairs Medical Center – including Andrew McNeal as first author – report that external rather than cell-intrinsic cues could be why mole melanocytes stop growing (McNeal et al., 2021).

The team analyzed existing datasets of individuals with either melanomas or moles, looking for gene expression differences. This revealed that certain micro-RNA (miRNA) transcripts, a class of non-coding RNAs involved in regulating gene expression, are present in higher levels in moles than in melanomas or healthy skin melanocytes.

To investigate if these differences are responsible for stopping cell division, McNeal et al. introduced the most highly elevated miRNAs into healthy skin melanocytes grown in cell culture. After seven days, the number of melanocytes containing either of two miRNAs was indeed lower compared to control melanocytes. On a closer look, cells treated with these miRNAs stopped dividing at the same phase of the cell cycle as moles, but not at the phase observed in senescent cells. The two miRNAs also inhibited the expression of a protein, known as AURKB, which is involved in the cell division process. Increasing the amount of AURKB in the miRNA-treated cultured melanocytes partially restored cell growth. An analysis of clinical mole and melanoma samples further showed that moles had lower levels of this protein than melanomas.

McNeal et al. also found when cultured melanocytes with activated *BRAF* stopped dividing, they still could resume growing once the oncogene was turned off, even after weeks of arrest. So, does the *BRAF* mutation simply stop division by upregulating miRNAs that (reversibly) block the cell cycle? One problem with this idea is that it does not explain why moles reach visible sizes, which requires an oncogene-expressing cell to divide many times before stopping.

For this and other reasons, McNeal et al. suspected that *BRAF* was only part of the story. They focused on a drug called TPA, which is usually added to melanocyte cell cultures because these cells usually grow poorly without it. Paradoxically, TPA had been reported to inhibit the proliferation of melanoma cells, suggesting its effects might depend on whether the *BRAF* gene is normal or mutated. Remarkably, simply eliminating TPA from the media used to culture *BRAF*-activated melanocytes stopped growth arrest and caused the cells to proliferate robustly. In fact, the experiments revealed that upregulation of one of the two miRNAs elevated in *BRAF*-activated melanocytes was mainly an effect of TPA itself, rather than *BRAF*.

A broader analysis of the individual and joint effects of TPA and *BRAF* suggested a model in which TPA acts on the differentiation state of melanocytes. TPA drives normal melanocytes from a quiescent, stem-cell like state to a proliferative one – which explains why TPA is needed in melanocyte cultures; however, in melanocytes driven to proliferate by activated *BRAF* the same mechanism leads to a miRNA-driven arrest in cell division (Figure 1).

**Figure 1.**
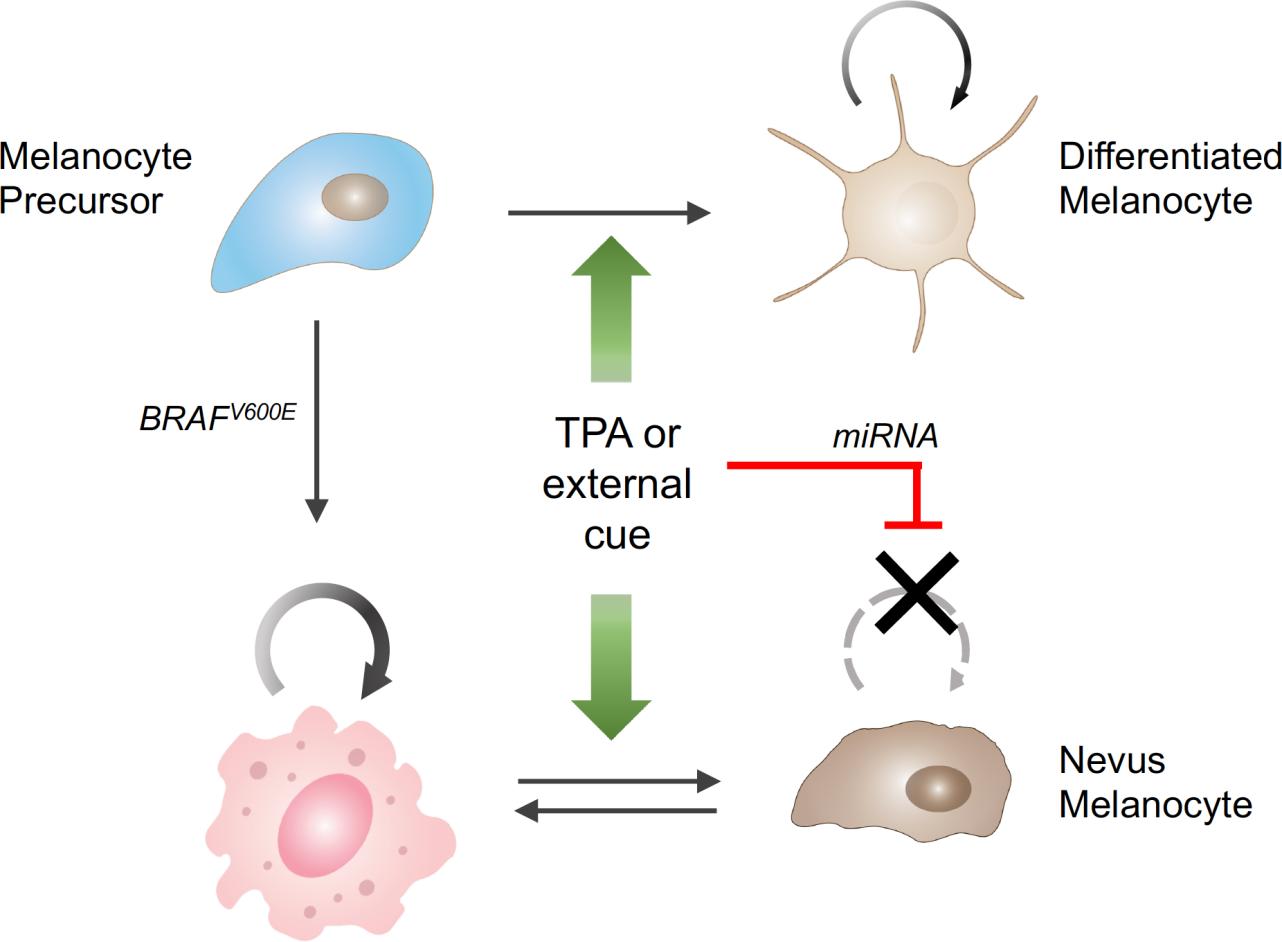
The influence of external stimuli on melanocytic cell growth. Healthy melanocyte precursors (light blue cell, top left) differentiate into melanocytes capable of proliferation (light brown cells, top right) under the influence of external signals, which can be mimicked in cell culture by the drug TPA (green arrows). However, when these precursors acquire a mutation in the *BRAF* gene (pink cell, bottom left), which initially stimulates them to divide, the same external signals ultimately cause them to stop dividing (brown cells, bottom right), through a process involving the upregulation of the production of certain miRNAs (red bar). The resulting collections of cells appear in the skin as pigmented moles.

The difference between normal and mutated cells does not seem to stem from the miRNAs themselves – they get upregulated in both cases – but from the fact that *BRAF*-activated melanocytes are much more dependent on AURKB. The reasons for this are unclear, but McNeal et al. showed that increasing AURKB levels in nevus melanocytes placed in tissue culture induced proliferation.

Together, these results suggest that the joint action of TPA and *BRAF*-activation on melanocytes in culture closely mimics the process that arrests moles in the body. Yet, since TPA is an artificial drug, McNeal et al. propose that this compound serves as a substitute for signaling molecules that normally come from skin cells around moles. This idea is consistent with recent work in mice suggesting that growth arrest in melanocytes cannot be an entirely cell-intrinsic process, but must involve extrinsic signals, such as growth-inhibitory proteins secreted by neighboring cells (Ruiz-Vega et al., 2020). The exact nature of such signals, and why they might appear only after moles have been growing for a while, remain topics for future research.

The work by McNeal et al. indicates that extracellular signals from tissues can be a determining factor in whether oncogenes drive cells toward or away from cancer. Better understanding the processes involved could help find new ways to stop skin cancers from growing, or even from developing in the first place.
